# How Realistic Is Threat Image Projection for X-ray Baggage Screening?

**DOI:** 10.3390/s22062220

**Published:** 2022-03-13

**Authors:** Robin Riz à Porta, Yanik Sterchi, Adrian Schwaninger

**Affiliations:** Institute Humans in Complex Systems, School of Applied Psychology, University of Applied Sciences and Arts Northwestern Switzerland, 4600 Olten, Switzerland; yanik.sterchi@fhnw.ch (Y.S.); adrian.schwaninger@fhnw.ch (A.S.)

**Keywords:** aviation security, X-ray imaging, human–machine interaction, X-ray baggage screening, threat image projection, visual search

## Abstract

At airports, security officers (screeners) inspect X-ray images of passenger baggage in order to prevent threat items (bombs, guns, knives, etc.) from being brought onto an aircraft. Because threat items rarely occur, many airports use a threat-image-projection (TIP) system, which projects pre-recorded X-ray images of threat items onto some of the X-ray baggage images in order to improve the threat detection of screeners. TIP is regulatorily mandated in many countries and is also used to identify officers with insufficient threat-detection performance. However, TIP images sometimes look unrealistic because of artifacts and unrealistic scenarios, which could reduce the efficacy of TIP. Screeners rated a representative sample of TIP images regarding artifacts identified in a pre-study. We also evaluated whether specific image characteristics affect the occurrence rate of artifacts. 24% of the TIP images were rated to display artifacts and 26% to depict unrealistic scenarios, with 34% showing at least one of the two. With two-thirds of the TIP images having been perceived as realistic, we argue that TIP still serves its purpose, but artifacts and unrealistic scenarios should be reduced. Recommendations on how to improve the efficacy of TIP by considering image characteristics are provided.

## 1. Introduction

As an integral part of aviation security, passenger baggage and other consignments are screened using X-ray machines at airport-security checkpoints in order to prevent threat items (bombs, guns, knives, etc.) from being brought onto an aircraft [1]. The X-ray images are inspected by airport-security officers (screeners), which involves a visual search and decision making [2]. This task is challenging for various reasons [3,4,5], one of which being that the low prevalence of threat items in X-ray images results in lower detection due to a shift in response tendency [6,7,8]. Threat image projection (TIP) is used at many airports worldwide to increase target prevalence by projecting pre-recorded images of threat items, also called fictional-threat images (FTIs), onto X-ray images of the baggage and other consignments being screened [9,10,11]. Moreover, with TIP, screeners receive frequent detection-performance feedback, which is otherwise missing because real threats rarely occur in practice. Performance feedback is an integral factor of job motivation [12,13]. The positive effect of feedback on motivation has also been shown to further translate into better cognitive performance [14,15], suggesting that TIP could also increase detection performance by frequently providing feedback. TIP is also used as an operational-performance measure [9,10,11]. Screener responses are recorded and summarized by calculating the proportion of detected TIP images (the hit rate). This hit rate is used to identify screeners with insufficient performance and trigger corrective actions. For example, at many airports, based on EU regulation [16], screeners who miss a minimum TIP hit rate must undergo remedial training and only resume screening after passing an X-ray-image-interpretation test. 

TIP is employed under the premise that TIP images look realistic. Therefore, they should not look different from the images of baggage containing real prohibited articles. However, based on interviews and observations in an ethnography study, Bassetti [17] reported that this is not always the case and that screeners recognize some TIP images because they look artificial. Depending on how often this occurs, it could constitute a serious problem as many airports worldwide use TIP scores as operational-performance measures. Moreover, TIP artifacts could also result in a security issue if screeners focus on detecting TIP artifacts instead of visually searching for prohibited articles in X-ray images of passenger baggage. We, therefore, conducted two studies to determine the prevalence of TIP artifacts. In a pre-study, we interviewed screeners to ascertain which artifacts occur in TIP images. In the main study, screeners rated a representative sample of TIP images regarding artifacts identified in the pre-study. In the main study, we also evaluated whether specific image characteristics affect the occurrence rate of artifacts.

## 2. Pre-Study

### 2.1. Method

#### 2.1.1. Participants

Nine screeners (four females and five males) participated in the pre-study. Their age was between 32 and 61 years (mean: *M* = 46.56, standard deviation: *SD* = 10.03), and they had 3 to 22 years of work experience (*M* = 9.00, *SD* = 5.68). Our studies (the pre- and main study) complied with the American Psychological Association Code of Ethics. They were approved by the institutional review board of the University of Applied Sciences and Arts Northwestern Switzerland. All pre- and main-study participants were qualified, trained, and certified according to the standards of the appropriate national authority (civil aviation administration) in compliance with the relevant EU regulation [16]. All participants provided informed consent in writing and were compensated according to their hourly rate of remuneration.

#### 2.1.2. Procedure

**Figure 1:**
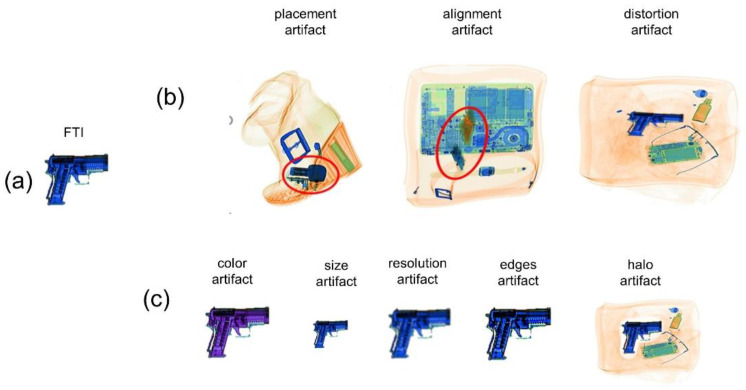
(**a**) Pre-recorded fictional-threat image. (**b**) Artifacts originating from an improper positioning of the FTI. (**c**) Artifacts originating from improper merging of the FTI with the X-ray baggage image. Note: the images with placement and alignment artifacts are real images from the TIP system investigated in our study. In these images, the projected bombs may be difficult to identify to the untrained eye. Therefore, we highlight them with red frames in the figure. The images with artifacts in Figure 1c were created using GIMP for illustration purposes.

Participants were individually invited to participate in semi-structured interviews with open-ended and close-ended questions. We first briefly explained what TIP artifacts are and asked the screeners to freely describe which artifacts they encountered during work. Next, we went through a list of potential artifacts. This list was created through discussions with screening experts before the interviews by considering how TIP artifacts could emerge when considering FTI TIP algorithms. Each artifact was explained to the participants and we asked whether they had noticed it during work. We started with three artifacts that might emerge when the FTI TIP system selects an FTI and then positions it in an unrealistic location or orientation in the X-ray image of the baggage (Figure 1b):

- Placement artifacts: The FTI is positioned such that it appears to penetrate an item in the baggage (e.g., the heel of a shoe).
- Alignment artifacts: The FTI is oriented such that it is poorly aligned with the content of the baggage. For example, the FTI is oriented at a 45° angle, while all other items are neatly packed and horizontally oriented. In some cases, the FTI can be oriented such that it appears to float (e.g., when an FTI bomb is oriented at a 45° angle away from a book or laptop lying flat).
- Distortion artifacts: This type of artifact refers to unrealistic distortion. It should be noted that X-ray images generally display a distorted image of the recorded items. The distortion depends on the location of an item in relation to the X-ray-beam source. The distortion of the FTI can appear unrealistic if it does not appropriately reflect the location in relation to the X-ray source.

Participants were first asked whether the FTI was sometimes in an unrealistic position. They were then asked to specify why the position was unrealistic and whether it was because of placement or alignment artifacts. Later, they were asked about distortion artifacts. Afterward, participants were questioned about artifacts that might emerge after the FTI had been selected and positioned. The FTI TIP system merges the FTI with the X-ray image of the baggage. For this step, the TIP system must ensure that the merged FTI has a realistic color and luminosity, which requires considering the material and density information of the projected item and the overlapping items of the X-ray image. Additionally, the TIP system must ensure that the FTI and the X-ray baggage image are identically processed (e.g., the identical edge enhancement is applied). Participants were asked about six artifacts that could result from the improper merging of the FTI with the baggage image (see Figure 1c):

- Color artifacts: The color of the FTI is unrealistic. For example, the color of an FTI gun looks different from the color of actual X-rays of guns.
- Size artifacts: The size of the FTI is unrealistic. The FTI is too small or too large.
- Resolution artifacts: The image resolution of the FTI differs from the image resolution of the other items in the TIP image.
- Edges artifacts: The edges of the FTI differ from the edges of the other items in the TIP image.
- Halo artifacts: There is a lightened area surrounding the FTI like a halo.

Finally, participants were also asked whether there were any indicators of TIP images outside the image itself, such as computer noises or a small lag from the additional processing.

#### 2.1.3. Results

**Figure 2:**
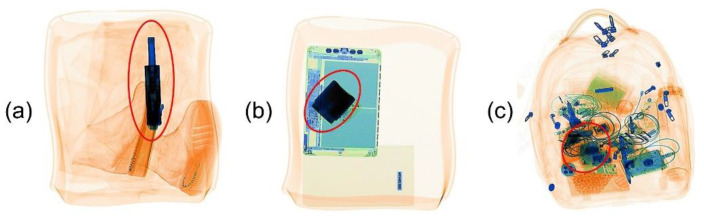
Example TIP images of unrealistic scenarios (**a**,**b**) and a TIP image depicting a realistic scenario (**c**). The projected gun (**a**) and the projected bombs (**b**,**c**) are highlighted with red frames.

While freely describing artifacts, screeners did not mention any artifact outside the list of potential artifacts (artifacts in Figure 1). Five out of nine participants reported that TIP sometimes improperly positions the FTI, causing placement and alignment artifacts (one participant while freely elaborating and four participants when asked specifically). Few participants reported other artifacts. Color artifacts were mentioned by one participant while freely elaborating and by one participant when specifically asked. Resolution artifacts were reported by one participant when specifically asked. The FTI size being sometimes too small was mentioned by two participants when feely elaborating, and the FTI size being sometimes too large was only mentioned by one participant. Issues with edges were mentioned by one participant when specifically asked. The halo artifact was never reported. In addition to artifacts, another form of unrealistic TIP images was reported: four screeners mentioned that even when no artifacts were present, TIP images frequently looked unrealistic because they showed an unrealistic scenario (see Figure 2). The most common case reported by participants was a threat item placed where no terrorist would hide it. For example, a gun is placed on top of a small purse, where it would be easily detected. Interestingly, screeners also reported that unrealistic TIP images (due to artifacts or unrealistic scenarios) occur more often when the FTI is projected onto loose items in a tray (e.g., trays with a shoe, jacket, wallet, or laptop) compared to when it is projected onto a bag or other piece of baggage (bag, suitcase, or rucksack). With regard to the question of whether there were any indicators of TIP images outside the image itself, such as computer noises or a small lag from the additional processing, all participants reported that this was not the case. 

#### 2.1.4. Discussion

**Figure 3:**
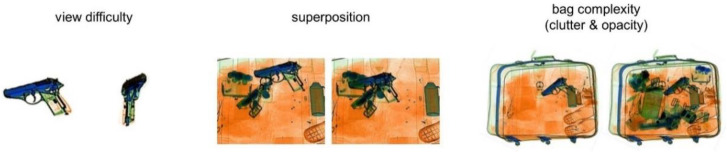
Illustrations of three image-based factors (IBFs). Adapted from Schwaninger et al. [18].

Most screeners reported placement and alignment artifacts. Color, size, resolution, distortion, and edge artifacts were only reported by a few. Different possible explanations exist as to why some artifacts were only reported by a few screeners. They might be rare, and not all participants might have encountered them. Most likely, screeners also differ in their assessment of what is realistic. For example, some participants mentioned that FTIs could be very large, which can seem strange. However, these items were large in reality (e.g., a machine gun), so the image was realistic despite seeming odd. When an object appears odd regarding its color, resolution, size, or edges, it might appear artificial to some screeners. Others judge it to be odd looking yet realistic. Screeners also reported that TIP images can look unrealistic because they depict an unrealistic scenario, even if they are free of artifacts (see Figure 2 for illustrations). Such unrealistic scenarios might, like artifacts, reduce the degree to which TIP as a performance measure reflects the detection of realistic threats (i.e., reduce the validity of TIP as a performance measure) and should therefore also be addressed. Moreover, whether unrealistic scenarios occur more often when an FTI is projected onto loose items in a tray (e.g., trays with a shoe, jacket, wallet, or laptop) compared to when the FTI is projected onto a bag or other pieces of baggage merits further investigation. Related to this issue is whether the perception of artifacts and unrealistic scenarios depends on image characteristics that have been shown to influence threat detection in X-ray-image inspection. Schwaninger et al. [18] identified three image characteristics, which they coined image-based factors (IBFs) and are illustrated in Figure 3. That is, a threat item can be more or less difficult to detect depending on its orientation (effect of view difficulty), the superposition of other items (effect of superposition), and the visual complexity of the bag (effect of bag complexity), which consists of clutter, the bag’s background unsteadiness, opacity, and the relative size of opaque areas in the bag [19]. These IBFs could have an impact on the perceived occurrence of artifacts and unrealistic scenarios.

## 3. Main Study

The primary aim of the main study was to determine the prevalence of the artifacts and unrealistic scenarios identified in the pre-study. Further, we wanted to analyze whether artifacts and unrealistic scenarios occur less often when the FTI is projected onto a piece of baggage versus onto loose items in a tray, and whether the perceived occurrence of artifacts and unrealistic scenarios is dependent on IBFs.

### 3.1. Method

#### 3.1.1. Participants

A total of 51 professional cabin-baggage screeners from a European international airport (29 females and 22 males) participated in the main study. None of them had participated in the pre-study. Their work experience ranged from 2 to 31 years (*M* = 8.67, *SD* = 4.91). The participants’ ages ranged from 25 to 63 years (*M* = 44.67, *SD* = 11.43). 

#### 3.1.2. Materials

We randomly sampled 600 TIP images (X-ray images of passenger bags with an FTI projected onto them) from the automated storage of 14 conventional single-view X-ray machines from the airport where the screeners worked. The content of the cabin baggage varied over the year. Therefore, we randomly selected half (300 images) of the X-ray images in winter, and the other half (300 images) in summer. The TIP hit rate is typically high; previous studies have reported values of about 90% [9,11] and approximately 80% [10]. We found an average TIP hit rate of 88% at the airport from which we sampled our images. A high TIP hit rate can result in low statistical power when computing correlations. To address this issue, missed TIP images were oversampled. A total of 75% of the images were sampled from hits and 25% (compared to 10%) from misses, totaling 450 detected and 150 missed TIP images that were used in the main study.

#### 3.1.3. Procedure

Participants attended two sessions that were two weeks apart with five to six screeners per session. The first session began with an hour-long introduction with visual illustrations of the different artifacts in order to impart the underlying concepts. This activity was followed by an explanation of how to use the rating tool and six practice trials. Participants then rated TIP images for two hours. They continued rating the images for another two hours during the second session. During both sessions, participants rated as many images as they could and were not incentivized to rush. They were instructed to take breaks when they felt fatigued. X-ray images were displayed on 24 inch Samsung S24E650BW monitors under normal lighting conditions at a distance of approximately 60 cm. The images covered approximately two-thirds of the screen corresponding to a visual angle of about 28 × 30 degrees. The participants were randomly split into four groups as it was not practically feasible for each screener to rate all 600 images. Each group rated a different set of 150 images, with the order of the images randomly sampled for each screener. After having rated all images of their group, the participants proceeded to rate the images of other groups if the sessions were not yet over. Ultimately, each of the 600 TIP images was rated by at least 12 participants.

#### 3.1.4. Measures

To collect the ratings from the participants, we developed an app using R-shiny [20] that consecutively displayed the TIP images. Participants could press a button to fade-in a red frame around the FTI if they were uncertain as to which threat item was projected. We used a seven-point scale for all ratings. The rating questions and anchors are shown in Table 1. For the artifacts that were mentioned by only a few screeners in the pre-study (distortion, color, resolution, size, and edges artifacts), the screeners were instructed to rate these artifacts if they thought they were present in an X-ray image. The absence of a rating was coded as 1. The ratings for all other image characteristics were mandatory. The screeners also had to provide other ratings that were not relevant to our study and reported in a conference proceeding [21].

The FTI view difficulty is defined as the difficulty to recognize the threat item in the depicted orientation independently of the X-ray baggage image [18]. However, the study participants had to rate the FTI view difficulty based on the TIP image with the FTI often superimposed by other items, which might have distorted the rating. Inspired by Schwaninger et al. [22], we therefore calculated an additional measure of FTI view difficulty: the rate at which the FTI was missed across all TIP images displayed at the airport for a year (Equation (1)). The FTI view difficulty was calculated based on the airport’s TIP reports and from at least 29 TIP events per FTI (*M* = 375.53, *SD* = 194.27).

(1) $$FTIvd=\frac{N_{Misses}}{N_{Projections}}$$

Equation (1). FTI view difficulty (*FTI_vd_*) equals the number of TIP events the FTI was missed (*N_Misses_*) divided by the number of TIP events the FTI was projected (*N_Projections_*).

To determine whether the FTI was projected onto a piece of baggage or onto loose items in a tray, screening experts who did not participate in the pre- or main study inspected and categorized the TIP images. 

#### 3.1.5. Analyses 

For each X-ray image, the mean ratings across screeners were calculated and then rounded to the nearest integer. In the next section regarding results, the relative frequencies of these averaged ratings are reported. The ratings were corrected for the oversampling of missed TIP images (see Section 3.1.2). Finally, to measure how strongly the screeners agreed with each other, we calculated the inter-rater reliability using intraclass correlation coefficients (ICCs, [23]).

### 3.2. Results

The ICCs indicated good to excellent [24] inter-rater reliabilities for the following image characteristics: artificial in general (0.72), placement artifacts (0.85), alignment artifacts (0.75), unrealistic scenario (0.84), FTI view difficulty (0.89), superposition (0.93), clutter (0.94), and opacity (0.89). Most images were not rated to show any artifacts regarding the FTI’s appearance (size, resolution, color, and edges) or any distortion artifacts. In the rare cases when a screener rated an image to show one of these artifacts, they were largely alone in their rating. Unsurprisingly, the ICCs indicated low [24] inter-rater reliability for these artifacts: color (0.26), size (0.23), resolution (0.27), distortion (0.04), and edges (0.12).

**Figure 4:**
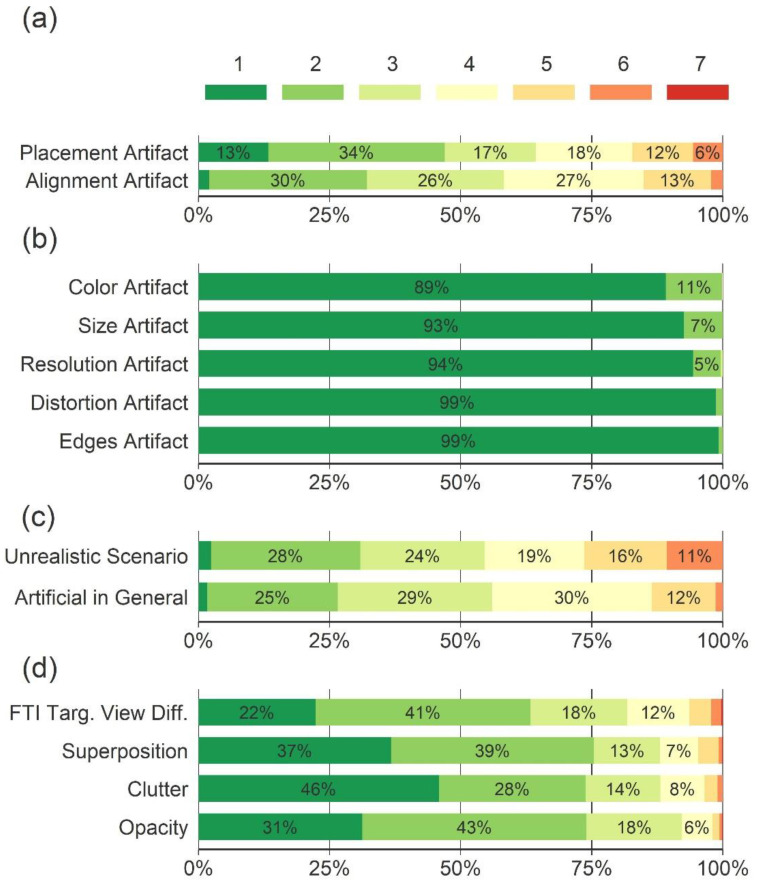
Results of the main study displayed as stacked bar charts. Each bar is divided into seven sub-bars displaying the share of TIP images that received the respective rating (averaged across participants). Regarding the presence of artifacts and unrealistic scenarios (**a**–**c**), participants selected ratings 1–3 to indicate that they disagreed that artifacts were present, 5–7 to indicate that they agreed that artifacts were present, and 4 to indicate that they were undecided. Regarding IBFs (**d**), ratings 1–7 represent low to high degrees of FTI view difficulty, superposition, and bag complexity in terms of clutter and opacity. Percentage numbers below 5% are not displayed.

Figure 4 shows how the TIP images were rated regarding artifacts and other image characteristics. In the figure, each bar is divided into seven colored sub-bars displaying the share of TIP images that received the respective rating. A total of 17% of the images received a rating indicating that placement artifacts were present (ratings of five or higher, Figure 4a), whereas 83% were rated neutral (rating four) or more toward the absence of a placement artifact (ratings one to three); 15% of the images received a rating indicating that alignment artifacts were present (Figure 4a). No image was rated to contain the artifacts that were only mentioned by a few screeners in the pre-study (color, size, resolution, distortion, and contour artifacts) with an average rating of five or above (Figure 4b). These artifacts were therefore excluded from further analyses. The screeners reported that 26% of the TIP images depicted an unrealistic scenario (ratings of five or higher, Figure 4c) and that 14% looked artificial in general. Figure 4d shows how the TIP images were rated regarding IBFs.

**Figure 5:**
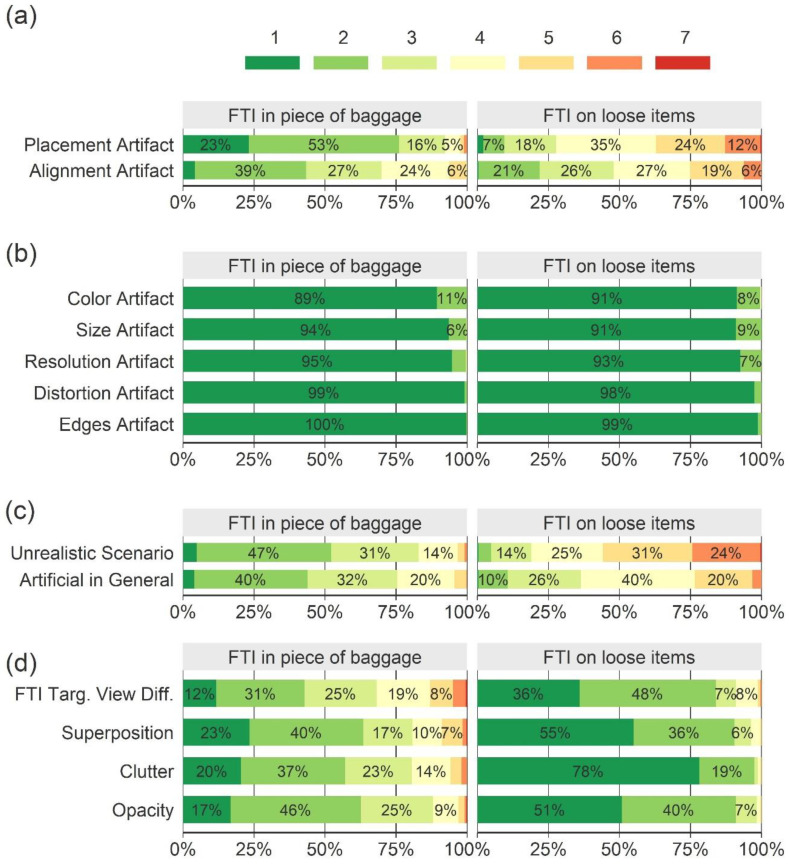
Results of the main study for the TIP images with the FTI in a piece of baggage and the TIP images with the FTI on loose items in a tray, displayed separately. Each bar is divided into seven sub-bars displaying the share of TIP images that received the respective rating (averaged across participants). Regarding the presence of artifacts and unrealistic scenarios (**a**–**c**), participants used ratings 1–3 to indicate that they disagreed that artifacts were present, 5–7 to indicate that they agreed that artifacts were present, and 4 to indicate that they were undecided. Regarding IBFs (**d**), ratings of 1–7 represent low to high degree of FTI view difficulty, superposition, and bag complexity in terms of clutter and opacity. Percentage numbers below 5% are not displayed.

Of all the TIP images, 42% were X-ray images with FTIs projected onto a piece of baggage (bag, suitcase, or backpack), and 58% were X-ray images with FTIs projected onto loose items in a tray. The two types of images were rated differently, as can be seen in Figure 5. In Table 2, the prevalence of different combinations of artifacts and unrealistic scenarios is reported. While 65% of all the TIP images with FTIs projected onto loose items in a tray showed at least either an artifact or an unrealistic scenario, this was only the case for 8% of all the TIP images with FTIs projected onto a piece of baggage.

For the TIP images with FTIs projected onto a piece of baggage, Table 3 depicts how IBFs correlate with placement and alignment artifacts and unrealistic scenarios. As can be seen, placement and alignment artifacts as well as unrealistic scenarios occur less frequently for higher values of IBFs. 

### 3.3. Discussion

No TIP image received an average rating indicating the presence of artifacts related to the FTI’s appearance (color, size, resolution, and edges) or distortion. If a participant rated an image to depict one of these artifacts, they mostly stood alone, confirming the finding of the pre-study that these artifacts are very rare or at least not perceived by most screeners. However, placement- and alignment-artifact ratings indicate that 17% of the TIP images contained placement artifacts, and almost as many images contained alignment artifacts (15%). A total of 24% were rated to contain at least one of the two artifacts and 26% of the TIP images were rated to depict an unrealistic scenario. Overall, 34% of the images were rated to depict an artifact or an unrealistic scenario. Fewer images were rated as artificial looking in general (14%) than as containing artifacts (24%), which at first might seem contradictory but could be due to a different focus. When screeners evaluated whether a TIP image looked artificial in general (which was asked first), the focus was likely broad, and no artifacts might have been apparent. When the screeners subsequently evaluated specific artifacts, the focus was narrowed, and the artifacts may have become evident.

Our results suggest that artifacts and unrealistic scenarios occur less often in images for which the FTI is projected onto a bag or other pieces of baggage. Only 7% of these images were rated to contain artifacts, only 3% to depict unrealistic scenarios, and only 8% were rated to contain artifacts or depict unrealistic scenarios. It is quite understandable that FTIs projected onto loose items were much more-often assessed as an unrealistic scenario, as it seems unlikely that terrorists would not try to hide a threat more effectively. Our findings also suggest that there are more degrees of freedom in merging an FTI with a piece of baggage without the placement or alignment looking unrealistic. TIP images with the FTI merged with a piece of baggage were rated to be more realistic when the FTI was superimposed by other items, when the baggage had high complexity, or when the view difficulty of the FTI was high. In an X-ray image, it is often impossible to see which objects are above or below each other. Images with higher complexity and superposition can therefore provide more degrees of freedom for realistic placement and alignment. Multiple possible explanations exist as to why FTIs with high view difficulty produce fewer artifacts. View difficulty is associated with the orientation of the FTI [18,25]. It also differs between different types of threats (bombs, guns, knives, etc.) [26,27,28]. Both of these aspects might, in turn, affect the prevalence of artifacts.

The FTI view difficulty was evaluated based on two different approaches: based on ratings or on the miss rate across all the TIP images with the same FTI. While the FTI view difficulty is defined as the difficulty of the FTI independently of the baggage image, the rated FTI view difficulty was correlated with superposition. Thus, raters perceived FTI view difficulty to be higher when superposition was higher, possibly because this made the FTI more difficult to detect. Therefore, it is not optimal to estimate view difficulty based on the rating of individual TIP images. Instead, the mean FTI-view-difficulty rating across several baggage images with different degrees of superposition should be taken, as has been done in previous studies [19,27,28,29]. Alternatively, the miss rate across many TIP images can serve as an estimate if sufficient TIP data is available [19,27,28].

## 4. General Discussion

Screeners visually inspect X-ray images of passenger baggage for prohibited articles, many of which rarely appear in reality. As rare targets are more challenging to detect, e.g., [6,7,8,30,31], threat image projection (TIP) could offer a solution by inserting pre-recorded images of threat items (fictional-threat images; FTIs) into randomly selected X-ray images of passenger baggage. As explained in the introduction, TIP has been associated with additional benefits, including the potential to increase motivation and performance by providing regular feedback, and measuring performance on the job. Bassetti [17] reported that screeners recognize some TIP images to be artificial, which could impair the efficacy of TIP. This study aimed to determine the prevalence of TIP-image artifacts. In the pre-study, we interviewed screeners about artifacts they encountered at work. The interviews also revealed that some TIP images are unrealistic because they display unrealistic scenarios. This refers to threat placements that terrorists would not use because they would be easy to find. In the main study, screeners rated a sample of 600 TIP images regarding TIP artifacts and unrealistic scenarios. Further, we evaluated whether certain image characteristics affect the occurrence of artifacts and unrealistic scenarios. 

### 4.1. Prevalence of Artifacts and Unrealistic Scenarios in TIP Images

A total 34% of TIP images were considered unrealistic because of artifacts or unrealistic scenarios, 24% were considered to contain an artifact, and 26% to display an unrealistic scenario. The main concerns regarding artifacts were unrealistic placement (17% of the TIP images) and unrealistic alignment of the FTI (15% of the TIP images). Other artifacts were rarely reported in the pre-study and were rarely rated to occur in the main study (unrealistic color, size, resolution, distortion, and edges of the FTI). Does the presence of artifacts and unrealistic scenarios impair the assumed benefits of TIP? Its most important application is to increase the frequency of rare threat items in order to enhance their detection [6,7,8]. This application relies on screeners detecting threat items rather than artifacts. Our results suggest that this requirement is still fulfilled for the majority of TIP images as 76% of the images were rated to be free of artifacts. Another application of TIP is to use the TIP hit rate for continuous performance evaluation [9,10,11]. If screeners do not achieve a minimum hit rate, they must undergo additional computer-based training and testing before they can resume screening duties [16]. As images with artifacts or unrealistic scenarios should generally be easier to detect than actual threats, the TIP hit rate is likely to overestimate the hit rate for real threats in real scenarios. The validity of TIP for screener comparison is supported by the finding that the better the screeners perform in TIP, the better they perform in a certification test that evaluates their threat-detection ability [32]. TIP hit rates should therefore be suitable as a performance measure for ergonomic or human-factors studies, which depend on the comparison of performances between screeners rather than on absolute performance [9,10,11]. 

In summary, we derive from our results that TIP is still useful despite the presence of TIP artifacts or unrealistic scenarios in about one third of the TIP images. However, the number of unrealistic images should certainly be reduced in order to increase the efficacy of TIP. With fewer unrealistic images, screeners would also perceive TIP hit rates as more-valid feedback on their job performance, which would likely make TIP more motivating. This assumption is consistent with work and psychology models that emphasize the importance of feedback for motivation and performance [12,13].

### 4.2. How to Reduce Artifacts and Unrealistic Scenarios

Our results suggest that artifacts and unrealistic scenarios occur less often in TIP images where the FTI is projected onto pieces of baggage (bags, suitcases, and backpacks). Only 7% of these TIP images were rated to contain artifacts and only 3% to depict unrealistic scenarios. Furthermore, 92% neither contained artifacts nor depicted unrealistic scenarios. In this investigation, TIP projected approximately one half of the FTIs onto pieces of baggage and the other half onto loose items such as shoes, jackets, wallets, keys, and laptops. Hence, TIP can be effectively enhanced by projecting threat images more frequently onto pieces of baggage. Our results further suggest that TIP images are more realistic when the IBFs view difficulty, superposition, and complexity are medium to high. The TIP system would require an algorithm to distinguish X-ray images of pieces of baggage from X-ray images of trays with loose items in them, and to estimate the IBFs of the TIP images to achieve fewer unrealistic TIP images; the latter, for example, with the algorithm developed by Bolfing et al. [27]. However, TIP should still project some FTIs onto images with loose items in a tray and lower IBFs in order to incentivize screeners to direct their attention towards these images. Otherwise, real threats might be missed due to a lack of focus. Another solution is offered by a different approach to TIP, in which baggage images and FTIs are combined in advance to create combined threat images (CTIs, [9]). These CTIs can be quality controlled to ensure that they do not exhibit any artifacts and show realistic scenarios. However, because CTIs are based on pre-recorded X-ray images of baggage, they can only be used in environments where screeners cannot directly see that the screened baggage does not correspond to the one shown on the TIP image. 

### 4.3. Limitations and Future Research

This study analyzed a common FTI TIP system for a single-view X-ray machine that is used at many airports. However, other FTI TIP systems are also used, which may differ regarding artifacts. Furthermore, we used TIP images from one airport. Baggage images from other airports might differ. For example, other airports may have more or fewer passengers with complex baggage. Future research should investigate artifacts and unrealistic scenarios in X-ray images from other TIP systems and airports. Newer X-ray machines show multiple views of the screened passenger baggage from different angles [33,34] or 3D-rotatable computed-tomography (CT) images [35]. This enhancement could provide further solutions for reducing artifacts in TIP. For example, based on the different views, a three-dimensional model of the baggage and its contents can be reconstructed [36,37,38] and used by the TIP system to find suitable positions and orientations for merging FTIs [39,40]. Our study is further limited by having screeners rate whether TIP images displayed artifacts. Therefore, we could only investigate artifacts that screeners could explicitly recognize. However, TIP images might also contain artifacts that screeners cannot consciously identify. TIP images could display artifacts that are not consciously perceived but subconsciously affect detection. Additionally, screeners only rarely see actual threat items (especially improvised explosive devices) and might not know precisely what they look like in an X-ray image. Therefore, they might have to infer from regular items (e.g., laptops, food, jackets, and keys) to determine whether rare threat items look artificial. This method is likely to work well for most artifacts (e.g., when the FTI is unrealistically aligned or when the FTI has different edges). However, it may not work for color artifacts because the color of the FTI could be off but may look realistic when solely compared to the color of regular items. 

## 5. Conclusions

Many airports use TIP to improve and measure the detection performance of screeners. The underlying assumption is that TIP is realistic. However, our study reveals that screeners consider every third TIP image unrealistic. While, these images are unlikely to render TIP ineffective, TIP systems should be improved through the more-frequent placement of FTIs inside actual pieces of baggage rather than onto loose items in a tray and more-often projecting FTIs onto baggage images with higher superposition and bag complexity.

## Figures and Tables

**Table 1:** Image characteristics, rating questions, and anchors. Rating questions and anchors were presented to participants in German and have been translated into English for the readers’ convenience.

Characteristics	Ratings	Anchors
Artifact		
Artificial in general	The X-ray image looks unrealistic because the FTI looks artificial.	Totally disagree (1) Totally agree (7)
Placement	The location of the FTI is unlikely.	“”
Alignment	The alignment of the FTI seems artificial.	“”
	Other reasons why the FTI seems artificial …	
Artifact		
Color	… due to unrealistic color	“”
Size	… due to unrealistic size	“”
Resolution	… due to unrealistic resolution	“”
Distortion	… due to unrealistic distortion	“”
Edges	… due to unrealistic edges	“”
Unrealistic Scenario	The TIP image looks unrealistic because the scenario is unrealistic.	“”
IBF		
FTI view difficulty	Difficulty to recognize the threat item in the depicted orientation	Very easy (1) Very difficult (7)
Superposition	Superposition of the FTI by other items	Very low (1) Very high (7)
Bag complexity clutter	Clutter in the baggage	Very low (1) Very high (7)
Bag complexity opacity	Proportion of the image that is opaque	Very small (1) Very large (7)

**Table 2:** Prevalence of artifacts, unrealistic scenarios, and combinations calculated both overall and separately for TIP images with FTIs projected onto a piece of baggage (bag, suitcase, or backpack) versus TIP images with FTIs projected onto loose items in a tray.

	Overall	FTI in Piece of Baggage	FTI on Loose Items in a Tray
Placement Artifact	17%	3%	37%
Alignment Artifact	15%	6%	25%
Any Artifact ^1^	24%	7%	45%
Unrealistic Scenario	26%	3%	56%
Any Artifact or Unrealistic Scenario	34%	8%	65%

^1^ At least one artifact rated to be present.

**Table 3:** Means (*M*), standard deviations (*SD*), Pearson correlations, and their 95% confidence intervals (in brackets) of ratings of placement and alignment artifacts, unrealistic scenario, and image-based factors for TIP images with the FTI in a piece of baggage.

Variable	*M*	*SD*	1	2	3	4	5	6
								
1. Placement Artifact	2.13	0.88						
								
2. Alignment Artifact	2.84	0.98	0.64 **					
			[0.57, 0.69]					
								
3. Unrealistic Scenario	2.66	0.89	0.81 **	0.69 **				
			[0.77, 0.84]	[0.64, 0.74]				
								
4. FTI View Difficulty (Rated)	2.98	1.28	−0.37 **	−0.48 **	−0.53 **			
			[−0.46, −0.28]	[−0.56, −0.40]	[−0.60, −0.45]			
								
5. FTI View Difficulty (TIP-Reports)	0.10	0.11	−0.17 **	−0.29 **	−0.29 **	0.48 **		
			[−0.27, −0.07]	[−0.39, −0.20]	[−0.39, −0.20]	[0.39, 0.55]		
								
6. Superposition (log)	0.78	0.47	−0.29 **	−0.32 **	−0.37 **	0.69 **	0.08	
			[−0.38, −0.19]	[−0.41, −0.22]	[−0.45, −0.27]	[0.63, 0.74]	[−0.03, 0.18]	
								
7. Bag Complexity (log)	0.81	0.45	−0.23 **	−0.18 **	−0.29 **	0.50 **	−0.01	0.73 **
			[−0.33, −0.13]	[−0.28, −0.08]	[−0.38, −0.19]	[0.42, 0.57]	[−0.11, 0.10]	[0.67, 0.77]
								

** *p* < 0.01.
